# Fermented Palm Kernel Cake Improves the Rumen Microbiota and Metabolome of Beef Cattle

**DOI:** 10.3390/ani14213088

**Published:** 2024-10-26

**Authors:** Wenbo Jiang, Yan Zhang, Haijian Cheng, Xin Hu, Wei You, Enliang Song, Zhiyong Hu, Fugui Jiang

**Affiliations:** 1 Key Laboratory of Livestock and Poultry Multi-Omics of MARA, Institute of Animal Science and Veterinary Medicine, Shandong Academy of Agricultural Sciences, Jinan 250100, China; a13561127723@163.com (W.J.); yanzi820106@126.com (Y.Z.); 98061107@163.com (H.C.); huxin19890803@163.com (X.H.); uv79@sina.com (W.Y.); enliangs@126.com (E.S.); 2 Shandong Provincial Key Laboratory of Livestock and Poultry Breeding, Jinan 250100, China; 3 College of Animal Science and Technology, Shandong Agricultural University, Taian 271018, China; hzy2004011@126.com

**Keywords:** fermented palm kernel cake, beef cattle, growth performance, microorganisms, metabolites

## Abstract

In this study, we utilised palm kernel cake as a substrate and fermented it with a composite of bacteria (*Pediococcus pentosaceus* CGMCC No. 27203 and *Lactobacillus plantarum* CGMCC No. 27202) and enzymes. We conducted a trial with twenty-four cattle, randomly divided into two groups of twelve cattle each. The control group (CON) was fed the standard farm diet, whereas the treatment group (PKC) received a diet with 3% of soyabean replaced by fermented palm kernel cake. The trial lasted for six weeks. The results showed no significant differences in growth performance between the PKC and CON groups. The abundance of *Firmicutes*, *Bacteroidetes*, *Proteobacteria*, and *Spirochaetes* was significantly higher in the PKC group than in the CON group. At the genus level, the abundances of *Anaeroplasma*, *norank_f__Bacteroidales_UCG-001*, *norank_f__Absconditabacteriales_SR1*, *norank_f__p-251-o5*, *Prevotellaceae_NK3B31_group*, *Prevotellaceae_UCG-001*, *Prevotellaceae_UCG-004*, and *Treponema* significantly increased in the PKC group. Lipid digestion and absorption pathways were significantly enriched in the PKC group. The results indicate that adding fermented palm kernel cake to the diet can increase the abundance of *Bacteroidetes* and *Fibrobacteres* in the rumen of beef cattle, enhancing the ability of the PKC group to degrade protein, carbohydrates, and fibrous materials in the feed, thereby improving the feed utilisation efficiency in beef cattle. Adding fermented palm kernel cake to the diet improved carbohydrate metabolism, metabolism of cofactors and vitamins, and nucleotide metabolism. Correlation analysis between the rumen microbiota and metabolic pathways showed that *Prevotellaceae_UCG-001* and *Prevotellaceae_UCG-003* were positively correlated with amino acid metabolism, *Rikenellaceae_RC9_gut_group* and *Succiniclasticum* were positively correlated with metabolism of cofactors and vitamins, and *Prevotella* and *Ruminococcus* were positively correlated with nucleotide metabolism. These findings elucidate the differences in rumen microbiota when fermented palm kernel cake is added to the diet, providing a theoretical basis for the application of fermented palm kernel cake in the diet of beef cattle.

## Simple Summary

In this paper, the effect of the dietary addition of fermented palm kernel cake on the structure and metabolites of the rumen microbiota of beef cattle was investigated. In this study, it was found that the addition of fermented palm kernel cake to the diet had no significant effect on the performance of beef cattle, enhanced their ability to decompose protein, carbohydrate, and fibre materials in the feed, and improved their feed utilisation. Notably, the fat digestion and absorption pathway was more pronounced in the group fed fermented palm kernel cake, enhancing the ability of beef cattle to regulate hepatic lipid metabolism, nutrient absorption, and energy metabolism. The addition of fermented palm kernel cake to the diet improved carbohydrate metabolism, cofactor and vitamin metabolism, and nucleotide metabolism. We believe these insights provide a theoretical basis for the use of fermented palm kernel cake in beef cattle diets.

## 1. Introduction

In recent years, the livestock industry has faced escalating production costs owing to factors such as African Swine Fever, the COVID-19 pandemic, and extreme weather conditions in certain regions. These challenges have led to shortages and price increases in feed supply, severely restricting the development of the livestock sector. To mitigate these issues, there has been a shift towards utilising inexpensive agricultural by-products and industrial residues as substitutes for traditional feed ingredients, such as soybean kernel cake. Among these alternatives, palm kernel cake, a by-product of palm oil fruit processing, is a promising option. Oil palms are extensively cultivated in tropical regions and yield significantly more oil per unit area than other oil crops [1]. According to the 2021 data from the Food and Agriculture Organization (FAO) of the United Nations, the global production of palm kernel cake amounted to 18.17 million tonnes.

Palm kernel cake is rich in crude protein (approximately 16%) and has a higher fat content than corn and soybean kernel cake, positioning it as an energy feed. Its nutritional profile is similar to that of corn gluten meal and its protein content is considered sufficient for most ruminants [2]. However, the presence of non-starch polysaccharides, primarily β-mannans, in palm kernel cake acts as an anti-nutritional factor, limiting the digestibility of nutrients by livestock [3]. Fermentation has been employed to degrade these anti-nutritional factors in palm kernel cake, thereby improving its nutritional value and feed utilisation and leading to cost-effective animal rearing.

Numerous studies have incorporated palm kernel cake into the diet of various livestock species. Umunna et al. demonstrated that including palm kernel cake in sheep feed did not adversely affect daily growth [4]. Jaworski et al. found that the addition of 15% palm kernel cake to the diet of weaned piglets did not affect their overall growth performance [5]. Ribeiro et al. showed that the addition of 19.5% palm kernel cake to the diet of lambs did not significantly alter the sensory quality of lamb meat [6]. Studies have also indicated that high-concentrate diets or diets lacking effective neutral detergent fibres can lead to increased endotoxin levels in the gastrointestinal tract of dairy cows [7], highlighting the importance of adding effective neutral detergent fibres to ruminant diets to improve gastrointestinal health. Alshelmani et al. observed that including fermented palm kernel cake in poultry diets improved the gut microbiota without affecting nutrient digestibility [8]. These advances underscore the viability of using palm kernel cake as a feed ingredient for ruminants.

The rumen hosts a complex microbial community that plays a crucial role in gastrointestinal health by digesting plant fibres into volatile fatty acids, proteins, and gases [9,10]. Feed is a key factor influencing the composition and function of rumen microbiota, altering the ruminal environment and metabolism [11]. For example, feeding Jerusalem artichokes increases the relative abundance of succinic acid-producing bacteria and rumen cocci, thereby enhancing ruminal fermentation and average daily weight gain [12].

Since 2020, China has banned antibiotics in feed, making fermented feeds a popular alternative because of their non-polluting nature, high utilisation rate, quality, and palatability. Thus, this experiment involved selecting suitable bacterial strains from palm kernel cake for fermentation, formulating beef cattle diets with high-quality fermented palm kernel cake, and conducting feeding trials. The use of 16S rRNA gene sequencing and metabolomics approaches can assess the effects of feeding on the rumen microbiome and metabolic functions of beef cattle before and after feeding. The results will help to elucidate the differences in the rumen microbiota of beef cattle after the inclusion of fermented palm kernel cake in their diets, providing a theoretical basis for its application in beef cattle diets.

## 2. Materials and Methods

### 2.1. Animals and Feeding Trial

In this study, we fermented palm kernel cake (Table 1) using a composite of microbes (*Pediococcus pentosaceus* CGMCC No. 27203 and *Lactobacillus plantarum* CGMCC No.27202) and composite enzymes (Table 2) to meet the nutritional needs of fattening cattle according to the cattle feed standards of China. Based on a large number of previous experiments, we finally concluded that the optimal fermentation conditions were 6 × 10^6^ cfu/g (*Lactobacillus plantarum* + *Pediococcus pentosaceus*) + 1 g/kg complex enzyme + 35% water + 65% palm kernel cake for 7 days.

Twenty-four Simmental crossbred cattle with Luxi Yellow cattle, with an average age of approximately 17 months and an average body weight of (628.95 ± 46.95) kg, were selected for this study. The cattle were randomly divided into two groups, each consisting of 12 beef cattle. The control group (CON) was fed a standard farm diet, while the treatment group (PKC) had 3% fermented palm kernel cake added to their diets. The trial feed was isoenergetic and isonitrogenous (Table 3).

The feeding trial lasted for six weeks. Feeding occurred at 06:00 and 18:00 daily, with leftover feed controlled to less than 10% (fresh weight). The cattle had free access to water throughout the day. The weights of all 24 fattening cattle were measured at the beginning and the end of the experiment.

After six weeks, four cattle from each group were randomly selected for slaughter after a 12 h fasting period. The eight cattle were humanely slaughtered in a commercial slaughterhouse (Shandong Lurun Co., Ltd., Dezhou, China). Within 20 min of slaughter and again within 30 min, approximately 2 g of rumen fluid was rapidly collected using 5 mL sterile cryogenic vials. These samples were immediately frozen in liquid nitrogen and stored at −80 °C for subsequent 16S rRNA gene sequencing and metabolomic analysis.

### 2.2. 16S rRNA Gene Sequencing and Data Processing

Genomic DNA from the total microbial community was extracted using the E.Z.N.A.^®^ Soil DNA Kit (Omega Bio-tek, Norcross, GA, USA), following the manufacturer’s protocol. The quality of the extracted DNA was verified by 1% agarose gel electrophoresis, and the concentration and purity were assessed with a NanoDrop2000 spectrophotometer (Thermo Scientific, Waltham, MA, USA). This DNA was then used as a template for PCR amplification of the V3-V4 regions of the 16S rRNA gene with primers 338F (5′-ACTCCTACGGGAGGCAGCAG-3′) and 806R (5′-GGACTACHVGGGTWTCTAAT-3′). [13], each carrying a barcode sequence. Each sample was amplified in triplicates. PCR products from the same sample were pooled and purified using the AxyPrep DNA Gel Extraction Kit (Axygen Biosciences, Union City, CA, USA) after electrophoresis on a 2% agarose gel, and quantified using a Quantus™ Fluorometer (Promega, Madison, WI, USA).

Quality control of the raw paired-end sequencing reads was conducted using Fastp software (version 0.19.6) [14] and the reads were merged using FLASH (version 1.2.11) software [15]. The merged sequences were clustered into operational taxonomic units (OTUs) at 97% similarity using UPARSE (version 7.1) software and chimeras were removed [16,17]. The OTU classification was performed against the Silva 16S rRNA gene database (v138) using the RDP classifier (version 2.11) with a confidence threshold of 70% [18]. The community composition of each sample was analysed at various taxonomic levels. Functional prediction of the 16S rRNA sequences was performed using PICRUSt2 (version 2.2.0) [19].

All data analyses were conducted on the Majorbio Cloud Platform (https://cloud.majorbio.com, accessed on 11 April 2023). Alpha diversity indices, including Chao and Shannon, were calculated using mothur software (version 1.30.2) [20], and group differences in alpha diversity were assessed via the Wilcoxon rank-sum test. Principal coordinate analysis (PCoA) based on the Bray–Curtis distance algorithm was used to evaluate similarities in microbial community structures across samples, with group differences analysed using PERMANOVA (version 1.1.0). LEfSe analysis [21] (LDA > 3, *p* < 0.05) was employed to identify bacterial taxa with significant differences in abundance between groups, from the phylum to genus level.

### 2.3. Metabolomics Analysis and Data Processing

The raw LC-MS data were processed using Progenesis QI (Waters Corporation, Milford, MA, USA) for baseline filtering, peak identification, integration, retention time correction, and peak alignment, resulting in a data matrix of retention times, mass-to-charge ratios, and peak intensities. Variables with a relative standard deviation (RSD) >30% in the QC samples were removed, and the data were log-transformed (base 10). The processed data matrix was then used for further analysis. MS/MS fragments spectra and isotope ratio difference with searched in reliable biochemical databases such as the Human metabolome database (HMDB) (http://www.hmdb.ca/, accessed on 12 April 2023) and Metlin database (https://metlin.scripps.edu/, accessed on 12 April 2023).

The preprocessed data were uploaded to the Majorbio Cloud Platform (https://cloud.majorbio.com, accessed on 13 April 2023) for analysis. Principal component analysis (PCA) and orthogonal partial least squares discriminant analysis (OPLS-DA) were performed using the R package ropls (version 1.6.2) with seven-fold cross-validation to assess model stability. The Student’s *t*-test and fold-change analysis were conducted to identify significantly different metabolites based on the variable importance in projection (VIP) scores from the OPLS-DA model and *p*-values from the t-tests (VIP > 1, *p* < 0.05). Metabolic pathways associated with significantly different metabolites were annotated using the Kyoto Encyclopedia of Genes and Genomes (KEGG) database. Pathway enrichment analysis was conducted using the scipy.stats Python package, and the pathways most relevant to the experimental treatments were identified through Fisher’s exact test.

### 2.4. Statistical Analysis

The experimental data were organised and computed using Excel 2016, and statistical analyses were conducted using R software (version 4.1.1). A one-way ANOVA was performed using the aov function. The standard error of the mean (SEM) was calculated using the lsmeans package, and multiple comparisons were conducted using Duncan’s method in the agricolae package. Statistical significance was set at *p* < 0.05.

## 3. Results

### 3.1. Growth Performance

**Figure 1:**
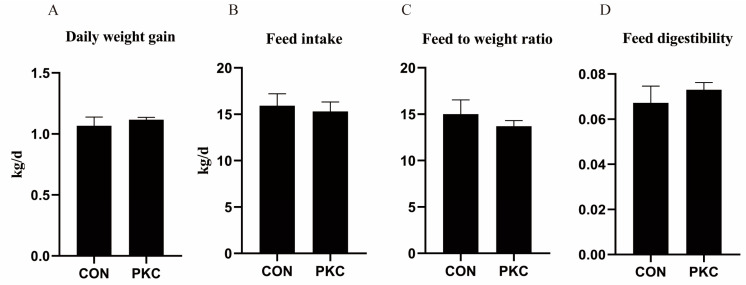
Effect of fermented palm kernel cake addition to the diet on growth performance of beef cattle. (**A**) Daily weight gain. (**B**) Feed intake. (**C**) Feed to weight ratio. (**D**) Feed digestibility. CON: Control group fed the standard farm ration; PKC: Group fed with 3% fermented palm kernel cake added to the standard ration.

The average daily weight gain, feed intake, feed-to-weight ratio, and feed digestibility of the two groups of cattle showed no significant differences (*p* > 0.05) (Figure 1A–D). However, the PKC group exhibited a certain degree of improvement in daily weight gain and feed efficiency.

### 3.2. Rumen Microbial Profile

**Figure 2:**
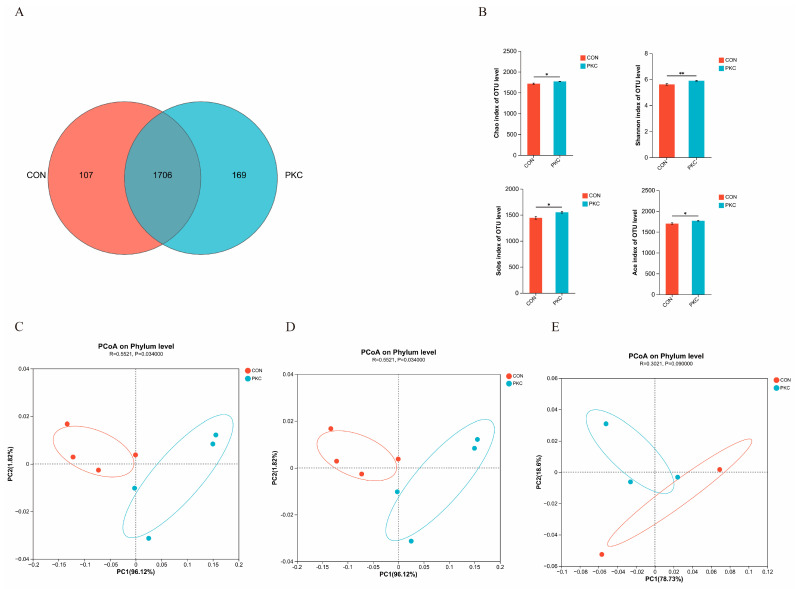
Effect of fermented mixed feed on rumen microbial composition. (**A**) Venn diagram showing the proportion of common and unique operational taxonomic units (OTUs) in each group. Alpha diversity indices, which represent diversity and evenness, are indicated by the Chao, Shannon, ACE, and Sobs indices (**B**). Beta diversity is analysed using non-metric multidimensional scaling (NMDS) based on the binary-Jaccard distance (**C**), principal coordinate analysis (PCoA) based on the unweighted Unifrac distance (**D**), and PCoA based on the weighted Unifrac distance (**E**). *, *p* < 0.05; **, *p* < 0.01; and ns, not significant.

In this study, we characterised rumen microbiota using 16S rRNA gene sequencing. After quality control and clustering, 1982 OTUs were identified, with 1706 OTUs common between the two groups. The CON and PKC groups contained 107 and 169 unique OTUs, respectively (Figure 2A). The alpha diversity of rumen microbial communities was assessed using the ACE, Chao, Sobs, and Shannon indices. Compared to the PKC group, the CON group showed a decrease in the ACE, Chao, Sobs, and Shannon indices (*p* < 0.05) (Figure 2B). Beta diversity, which reflects the similarity in microbial composition, was analysed using non-metric multidimensional scaling, PCoA based on weighted and unweighted UniFrac distances, and unweighted pair group method with arithmetic mean clustering (Figure 2C–E). These analyses revealed that the inclusion of fermented palm kernel cake in the diet had a large effect on microbial composition.

**Figure 3:**
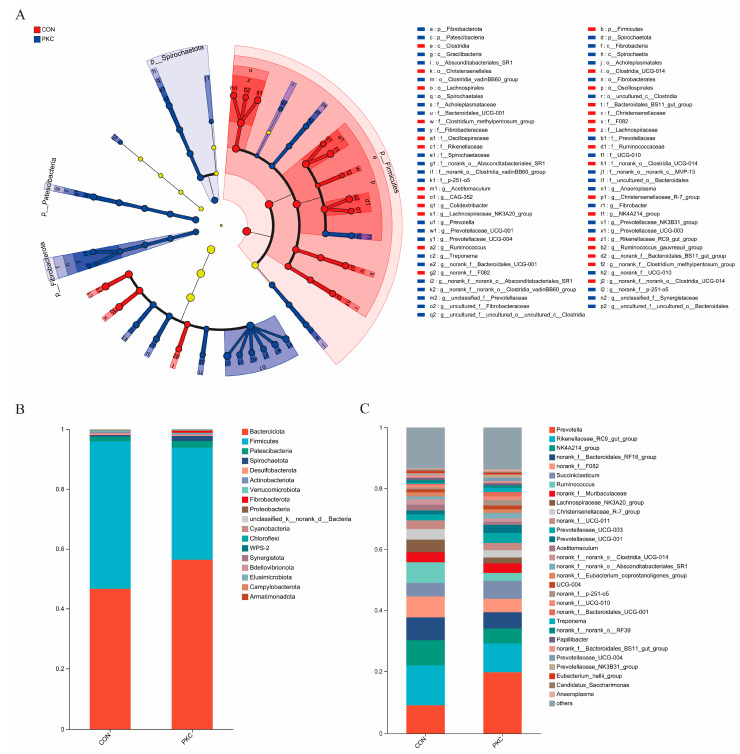
Effect of fermented mixed feed on rumen microbial communities. (**A**) Biomarker species identified by LEfSe analysis (*p* < 0.05, LDA > 3). (**B**) Top 18 genera. (**C**) Top 30 genera.

LEfSe analysis revealed 61 species with significant differences between the two groups (*p* < 0.05, LDA > 3) (Figure 3A). The CON and PKC groups contained 13 and 18 significantly enriched genera, respectively. For example, genera within the phylum Firmicutes, such as Ruminococcaceae and Escherichia, showed a significantly increased relative abundance in the CON group, whereas phyla such as *Patescibacteria*, *Fibrobacteres*, and *Bacteroidetes* and genera such as *Prevotella* were significantly more abundant in the PKC group.

To further clarify the composition of rumen microbial communities between the two groups, we analysed the relative abundance of microbial communities at the phylum and genus levels. The top 20 microbial taxa at the phylum level are shown in Figure 3B, with *Bacteroidetes* and *Firmicutes* as the dominant phyla. As shown in Figure 3C, at the phylum level, seven phyla showed significant differences between the groups. The PKC group exhibited significantly higher relative abundances of *Bdellovibrionota*, *Elusimicrobiota*, *Fibrobacteres*, *Patescibacteria*, *Proteobacteria*, *Verrucomicrobia*, and *Spirochaetes* than the CON group.

### 3.3. Rumen Metabolic Characteristics

**Figure 4:**
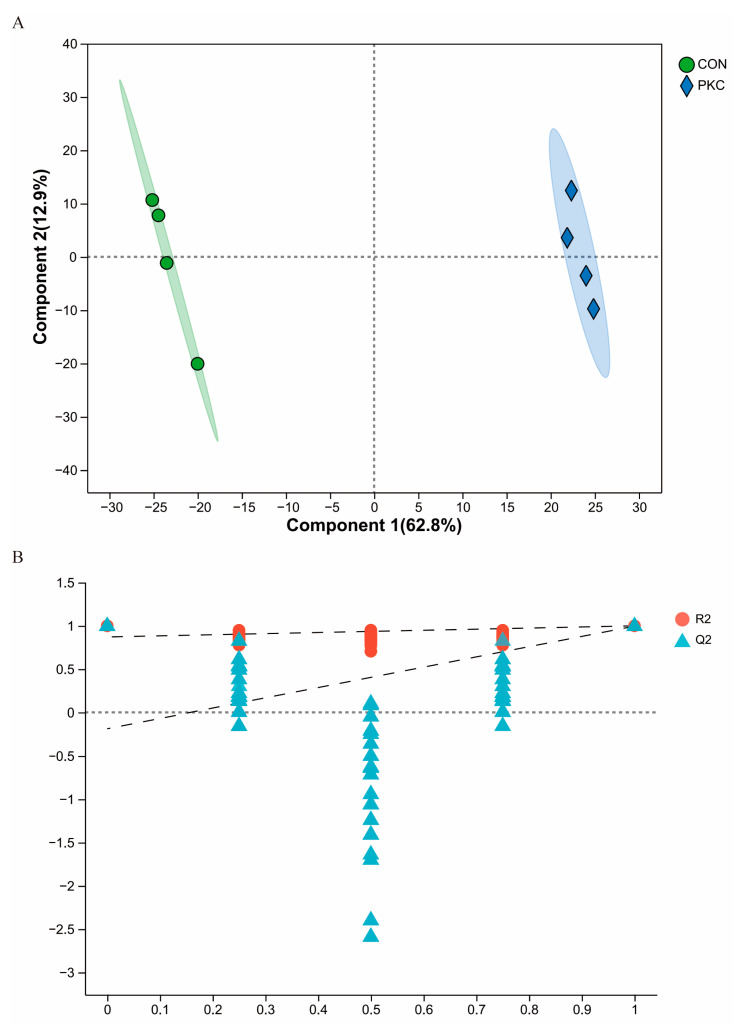
Effect of fermented mixed feed on rumen metabolite profiles. (**A**,**B**) Score plots from partial least squares discriminant analysis (PLS-DA) showing the separation of rumen metabolites between the CON and PKC groups.

To characterise the changes in metabolic products induced by palm kernel cake, we conducted an untargeted metabolomic analysis using LC-MS/MS. The aim of this analysis was to identify the spectra of the rumen metabolic products. To examine the differences in the composition of each group, a partial least squares discriminant analysis (PLS-DA) plot was used to elucidate metabolic patterns. The PLS-DA plot showed a clear separation between the two groups, indicating that the inclusion of fermented palm kernel cake in the diet significantly altered the rumen metabolic profiles (Figure 4A).

A permutation test was conducted to assess the validity and robustness of the PLS-DA model. The results showed that the R² values ranged from 0 to 1, and the cross-validated Q^2^ values ranged from 0 to 0.996. These metrics suggested that the model performed excellently in explaining data variability and predicting new data, confirming that the model was a good fit for the true state of the sample data (Figure 4B).

**Figure 5:**
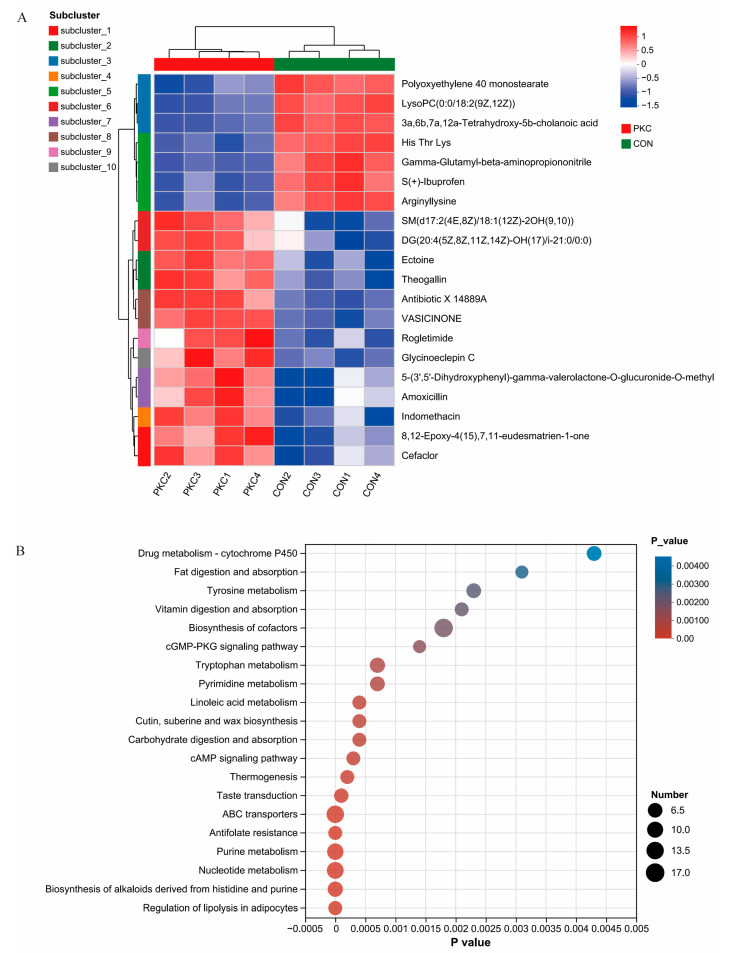
(**A**) Top 20 significantly different metabolites selected by ascending *p*−value and displayed through a heat map to illustrate differences in metabolite clustering. (**B**) KEGG pathway analysis of differences in metabolites between the two groups.

The top 20 most significantly different metabolites were selected according to ascending *p*-values and displayed in a heat map to show their differences in metabolite clustering (Figure 5A). Similar expression patterns may indicate synergistic actions or similar regulatory mechanisms of metabolites in biochemical pathways. In particular, metabolites that were generally expressed at higher levels in the PKC group, such as polyoxyethylene 40 monostearate and LysoPC (0:0/18:2(9Z,12Z)), suggest that the inclusion of fermented palm kernel cake in the diet modulates lipid metabolism and the composition and function of cell membranes, which are linked to inflammatory responses, cell signalling, and membrane structure maintenance.

To further investigate the specific effects of fermented palm kernel cake on metabolic pathways, KEGG pathway analysis was used to annotate the metabolic pathways involved. The top 20 enriched pathways were primarily associated with drug metabolism (cytochrome P450) and fat digestion and absorption (Figure 5B).

**Figure 6:**
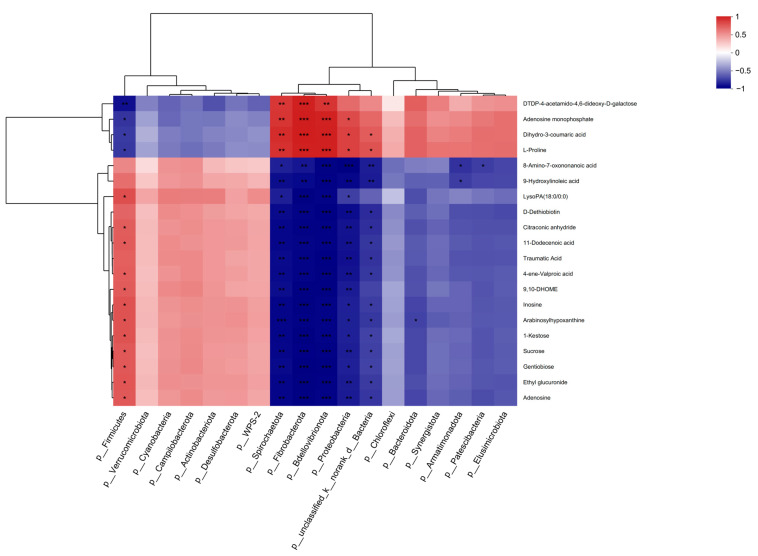
Spearman correlations between significantly different metabolites and bacterial taxa at the genus and phylum levels. Blue and red colors indicate negative and positive correlations, respectively. *, *p* < 0.05; **, *p* < 0.01; ***, *p* < 0.001.

Correlation analysis between the differential microbial communities and metabolites (Figure 6) showed that most of the differential metabolites were closely related to differential microbial taxa. Adenosine monophosphate showed a strong positive correlation with *Proteobacteria*, implying that an increase in the abundance of *Proteobacteria* was associated with elevated adenosine monophosphate levels. This indicates that the addition of fermented palm kernel cake to the diet modulates the rumen microbial community and metabolism.

### 3.4. Prediction of Rumen Microbial Function

**Figure 7:**
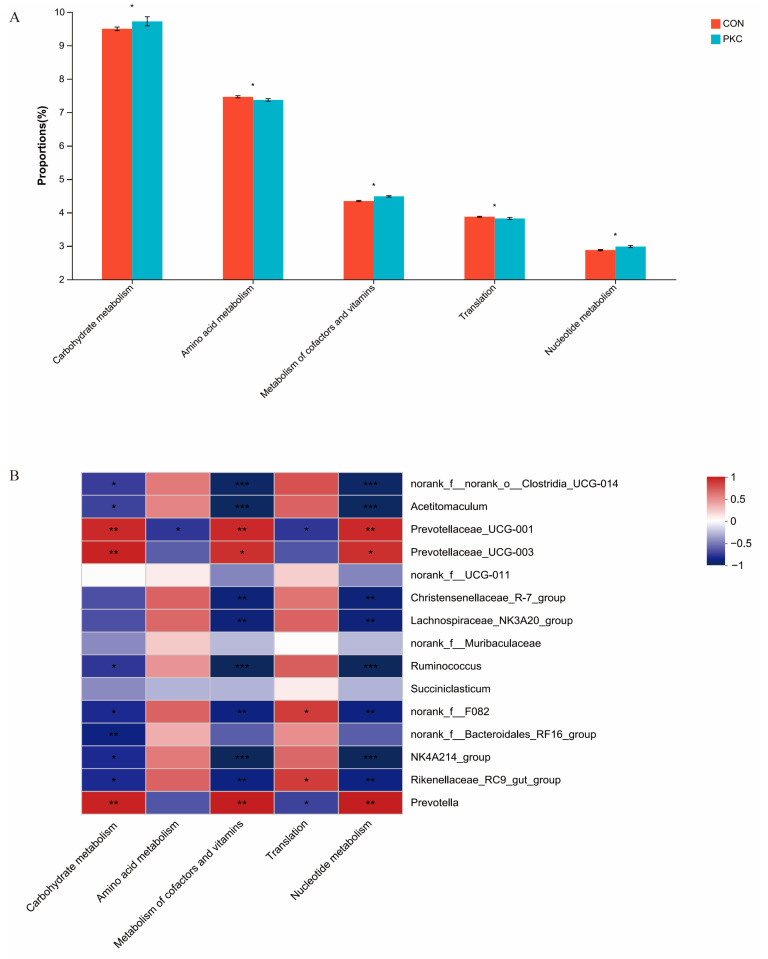
(**A**) Bar chart of predicted abundance for the PICRUSt2 function. (**B**) Heat map representation showing bacterial diversity and its associated predicted functional pathways. *, *p* < 0.05; **, *p* < 0.01; ***, *p* < 0.001.

To understand the metabolic functions of rumen bacteria, PICRUSt (version 2.2.0) software was used for functional prediction (Figure 7A). The metabolic pathways of carbohydrate metabolism, metabolism of cofactors and vitamins, and nucleotide metabolism in the PKC group showed significantly higher activity compared to the CON group, while the metabolic pathways of amino acid metabolism and translation in the PKC group showed reduced activity. Correlation analysis between differential microbiota and metabolic pathways (Figure 7B) indicated that carbohydrate metabolism was strongly positively correlated with *Ruminococcus* and *Prevotella*. *Prevotellaceae_UCG-001* and *Prevotellaceae_UCG-003* significantly promoted amino acid metabolism. *Rikenellaceae_RC9_gut_group* and *Succiniclasticum* significantly promoted the metabolism of cofactors and vitamins. Nucleotide metabolism showed a strong positive correlation with *Prevotella* and *Ruminococcus.*

## 4. Discussion

This study revealed no significant differences in the average daily weight gain, feed intake, or feed-to-gain ratio between the two groups of beef cattle. These results align with those of Chanjula et al., who observed that incorporating various proportions of palm kernel cake into goat feed did not significantly affect feed intake, suggesting that the inclusion of a certain proportion of palm kernel cake in beef cattle diets did not affect production performance [22].

Ruminal microbes play a critical role in converting indigestible feed into nutrients, and their species diversity and abundance are crucial for maintaining normal physiological functions [23]. Indices such as ACE and Chao reflect species richness, whereas the Shannon and Sobs indices indicate both richness and evenness. The results showed significant differences in these indices between the PKC and CON groups, with the PKC group exhibiting higher bacterial richness and diversity in rumen fluid. This increase is likely owing to the fermentation of palm kernel cake, which produces a large number of probiotics, enhancing microbial diversity and richness in the rumen.

The dominant phyla in the rumens of both cattle groups were *Bacteroidetes* and *Firmicutes*, consistent with the findings of Sim et al., who suggested that the dominant microbial communities in the bovine rumen were not affected by dietary structure [24]. *Bacteroidetes*, which is capable of breaking down proteins, carbohydrates, and fibrous materials in feed, was significantly more abundant in the PKC group, indicating that replacing part of the diet with fermented palm kernel cake enhanced the ability of cattle to decompose fibrous materials. Chen et al. demonstrated that higher hay content in the diet increased the abundance of Firmicutes, which can degrade structural polysaccharides [25]. The higher abundance of Firmicutes in the CON group, which had higher yellow storage content than the PKC group, supports this finding.

In this study, nine phyla exhibited the highest abundance in each treatment group: *Bacteroidetes*, *Firmicutes*, *Patricibacteria*, *Spirochaetes*, *Desulfovibrionaceae*, *Actinobacteria*, *Verrucomicrobia*, *Fibrobacteres*, and *Proteobacteria*. Notably, the PKC group showed a significantly higher relative abundance of *Spirochaetes* and *Fibrobacteres* than the CON group. Consistent with this study, Chen et al. found that the relative abundance of Spirochaetes increased with higher levels of dietary concentrate, whereas the content of corn stover silage decreased and the concentrate ratio increased [26]. Amin also found that the addition of fermented feed to the diet increased the relative abundance of Fibrobacteres and Spirochaetes in dairy cow rumen fluid, suggesting that the addition of fermented feed enhanced the breakdown of cellulose in beef cattle, which is consistent with the findings of this experiment [27].

At the genus level, the relative abundance of *Prevotella* in the rumen was significantly higher in the PKC group than in the CON group. *Prevotella* is a major bacterium within the *Bacteroidetes* phylum and is responsible for the breakdown of proteins, carbohydrates, and fibrous materials in feed. Replacing part of the diet with fermented palm kernel cake can increase the relative abundance of rumen *Prevotella* and enhance the digestion and absorption of proteins, carbohydrates, and fibre in beef cattle. The *NK4A214_group* and *Christensenellaceae_R-7*, both belonging to Firmicutes, play important roles in the degradation of cellulose and hemicellulose in the rumen [28,29]. The relative abundances of *NK4A214_group* and *Christensenellaceae_R-7* were significantly higher in the CON group than in the PKC group, consistent with the findings of Chen et al. [27]. A high-fat diet increases the relative abundance of *Rikenellaceae_RC9* and short-chain fatty acids in the body, leading to enhanced energy metabolism. Thus, the abundance of *Rikenellaceae_RC9* may be related to the short-chain fatty acids in the body [30]. This finding is consistent with that of Lan et al., who observed that an increase in *Rikenellaceae_RC9* in the body was correlated with an increased production of short-chain fatty acids in faecal microbial metabolic products. The rumen contains a complex microbial ecosystem that is particularly active during lipid metabolism [31]. Conte et al. showed that certain bacterial genera may significantly affect the presence of various fatty acids [32], and Liu found a strong positive correlation between *Lachnospiraceae_NK3A20* and the compound DMA18:1c12 [33]. Wang et al. discovered that when the intestinal health of the body is optimal, the abundance of short-chain fatty acid-producing bacteria, such as Colidextribacter, increases, thereby maintaining normal lipid metabolism [34]. High- and low-fibre diets influence the intestinal microbial community; *NK4A214* is the main bacterial genus involved in cellulose breakdown and its abundance affects carbohydrate, amino acid, and energy metabolism [35]. Yi et al. found that changes in the fine-to-rough feed ratio significantly affected the bacterial composition of yak rumen. A higher fine-to-rough feed ratio increased the relative abundance of F082, indicating that it may play an important role in carbohydrate digestion [36]. Additional research has found that supplementation with feed concentrate reduces the diversity of the ruminal bacterial community, such as a significant reduction in the relative abundance of *Absconditabacteriales_SR1* after concentrate supplementation [24], which is consistent with the results of the present study.

Mu et al. discovered that genera such as *Spirochaeta*, *p-251-o5*, and *Christensenellaceae_R-7* participate in cellulose degradation [37]. Conte et al. found that *Prevotellaceae_UCG-001*, *Prevotellaceae_NK3B31*, and *Prevotellaceae_UCG-004* are highly correlated with DMAC15:0, DMAC15:0iso, and DMAC17:0, respectively [30]. Clemmons et al. revealed that transitions from grass- to concentrate-based diets caused changes in the abundance of orders, such as *Bacteroidales*, *Pasteurellales*, and *Aeromonadales*, in the rumen. When dietary changes occur, specific bacterial populations in the rumen also change [38].

Metabolomics plays a crucial role in the study of beef cattle rumens. This study analysed the rumen metabolome to understand the differences in metabolic products under different diets, thus inferring the metabolic status of the rumen microbiome. The primary enriched metabolic pathways were drug metabolism involving cytochrome P450 and fat digestion and absorption. Specific cytochrome P450 enzymes exhibit varying abundance at different postpartum times, suggesting that they may play roles in regulating liver lipid metabolism and other co-metabolic pathways [39]. Furthermore, dietary changes in calves enhance fat digestion and absorption pathways, highlighting their important role in the development of the calf rumen, particularly in nutrient absorption and energy metabolism [40]. Overall, the rumen fluid of the PKC group contained microbial communities with significant drug metabolism (cytochrome P450) and fat digestion and absorption pathways, enhancing the ability of cattle to regulate liver lipid metabolism, nutrient absorption, and energy metabolism.

It has been reported that amino acid metabolism promotes bacterial growth and activity by providing sources of carbon and energy [41]. Carbohydrate metabolism may produce various compounds through the degradation of cellulose and hemicellulose [42]. The metabolism and function of vitamins and coenzymes in the body are crucial for maintaining normal physiological functions, as both deficiency and excess can lead to health problems. Pitta et al. found that when beef cattle were fed low-protein, high-fibre forage, which could convert high-protein, highly soluble nutrients from winter wheat, the proportion of *Prevotella* increased from 28% to 56%, making it the most predominant genus in the rumen. This genus is associated with protein degradation, similar to the results of this study; therefore, the predicted functions are also focused on amino acid transport and metabolism related to high-protein diets. KEGG primary metabolic pathway analysis showed that genes are mainly enriched in metabolism; secondary metabolic pathway analysis indicated that genes are primarily enriched in amino acid metabolism and carbohydrate metabolism within metabolism, which is consistent with the predicted amino acid metabolism and carbohydrate metabolism functions of this study. Cui et al.’s research demonstrated that feeding beef cattle whole-crop maize silage increased the abundance of *Prevotella_1*, improving carbohydrate metabolism, metabolism of cofactors and vitamins, and nucleotide metabolism pathways, which aligns with the results of this study [43]. Enrichment of the cytochrome P450 pathway suggests that cells may be actively processing drugs and other xenobiotic compounds. The significant enhancement of the fat digestion and absorption pathway in the PKC group indicates the activation of fatty acid metabolism and absorption processes, which are crucial for energy balance and lipid metabolism regulation.These findings indicate alterations in the rumen microbiota, along with improvements in carbohydrate metabolism, cofactor and vitamin metabolism, and nucleotide metabolism, which contribute to enhanced growth performance and rumen fermentation in beef cattle.

This study revealed the effects of adding fermented palm kernel cake to the diet on the microbial community and metabolites in the rumen of beef cattle. However, 16S rDNA sequencing and metabolomics alone cannot fully elucidate the effects of fermented palm kernel cake addition to beef cattle diets. In future research, we plan to study blood markers and volatile fatty acids in the rumen of both cattle groups and, combined with multi-omics analysis methods, provide robust evidence for the experimental results.

## 5. Conclusions

In summary, the inclusion of fermented palm kernel cake in the diet had no significant effect on beef cattle production performance. However, it enhanced the richness of *Bacteroidetes* and *Fibrobacteres* in the rumen. These bacterial communities increase the ability of cattle to break down proteins, carbohydrates, and fibrous materials in the feed, thus improving feed utilisation rates. Additionally, changes in the structure of the rumen microbial community led to alterations in rumen metabolites. Notably, the pathway for fat digestion and absorption was highly pronounced in the PKC group, enhancing the capacity of cattle to regulate liver lipid metabolism, nutrient absorption, and energy metabolism. PICRUSt functional prediction analysis showed that the dietary addition of fermented palm kernel cake group was rich in genes related to carbohydrate metabolism, cofactor and vitamin metabolism, and nucleotide metabolism. These findings provide useful information for the application of palm kernel cake in beef cattle.

## Figures and Tables

**Table 1:** Nutritional composition of palm kernel cake and fermented palm kernel cake.

Items	CP	Ash	EE	NDF	ADF
Palm kernel cake	16.88	4.26	7.83	71.90	12.17
Fermented palm kernel cake	17.48	4.16	6.86	53.63	5.30

CP, crude protein; Ash, crude ash; EE, ether extract; NDF, neutral detergent fibre; ADF, acid detergent fibre.

**Table 2:** Enzymatic profile of complex enzymes used in fermented palm kernel cake.

Items	Xylanase	Dextranase	Mannase	Cellulase	Pectinase	Protease
Enzyme, U/g	16000	3200	4000	800	1200	2000

**Table 3:** Composition and nutrient content of the test diets.

Items	CON	PKC
Ingredients, % of DM		
Maize	40.50	39.60
Soybean meal	5.40	4.70
Wheat bran	3.20	3.80
Fermented palm kernel cake	0	3
Baking soda	1.50	1.50
Calcium hydrogen phosphate	0.60	0.60
Calcium carbonate	0.60	0.60
Salt	0.60	0.60
Premix	0.60	0.60
Yellow storage	47.00	45.00
Nutrient content, % of DM		
CP	10.45	10.45
EE	3.31	3.35
NDF	38.39	39.12
ADF	26.23	26.61
Ash	6.58	6.46
Starch	33.08	32.55
Ca	0.22	0.22
P	0.34	0.35
ME Mcal/kg	2.34	2.34
NEm Mcal/kg	1.46	1.46
NEg Mcal/kg	0.88	0.88

CP, crude protein; EE, ether extract; NDF, neutral detergent fibre; ADF, acid detergent fibre; Ash, crude ash.

## Data Availability

The 16S rRNA sequence data were submitted to the NCBI Sequence Read Archive (SRA; https://submit.ncbi.nlm.nih.gov/subs/sra/, accessed on 16 May 2023) database with the accession number of PRJNA1112359 for open access.
